# Study on the Potential Mechanism of Astragaloside IV on Renoprotection in Db/Db Mice via Network Pharmacology and Experimental Validation

**DOI:** 10.1155/jdr/5345971

**Published:** 2026-03-31

**Authors:** Han Li, Baiju Wang, Hanwen Chen, Yuan Li, Zihua Song, Yajuan Chen, Xiaobing Li, Lei Liu, Na Wang

**Affiliations:** ^1^ Department of General Medicine, Affiliated Hospital of Jining Medical University, Jining, Shandong, China, jnmc.edu.cn

**Keywords:** AS-IV, DN, inflammation, network pharmacology, TNF-*α* signaling

## Abstract

**Background/Aims:**

Diabetic nephropathy (DN) is a highly predominant and critical microvascular complication associated with diabetes mellitus. Astragaloside IV (AS‐IV), the main active component in Radix Astragali, is associated with various pharmacological effects, including on DN. Nevertheless, the fundamental mechanism through which AS‐IV ameliorates DN is still unclear. Therefore, this research seeks to explore the molecular mechanisms underpinning the therapeutic effectiveness of AS‐IV for DN using network pharmacology and experimental validation.

**Methods:**

Initially, DN mouse models were created and administered AS‐IV treatment. Key metabolic parameters were assessed, and pathological alterations in the mice′s kidneys were examined. Subsequently, the GEO database and other online public databases were utilized to detect DN‐correlated targets and the primary AS‐IV targets. A protein–protein interaction (PPI) network of overlapping targets was created to pinpoint core targets using Cytoscape software. These core targets underwent GO and KEGG analyses. Differential gene analysis and ROC curve analysis were then conducted on the core targets. Finally, western blotting was conducted to confirm the expression changes of core targets and pathway proteins.

**Results:**

AS‐IV treatment markedly reduced fasting blood glucose (FBG), blood urea nitrogen (BUN), body weight (BW), urinary albumin excretion (UAE), and serum creatinine (Scr) levels and alleviated histopathological alterations in the DN mice′s kidneys. Twelve key core targets were identified through GEO data analysis and network pharmacology, with four core targets (MMP‐9, MPO, IL‐6, and IL‐1*β*) emerging after more stringent screening. Enrichment analysis illustrated that these genes predominantly participated in biological processes, such as regulating oxidative stress and inflammatory responses, and were part of pathways like TNF, IL‐17, and AGE‐RAGE signaling in DM complications. Western blotting further displayed that AS‐IV treatment downregulated the levels of DN‐affected proteins MMP‐9, MPO, IL‐1*β*, IL‐6, and TNF‐*α*.

**Conclusions:**

Overall, combining the network pharmacology and experimental validation offers a precise elucidation of the molecular mechanism by which AS‐IV treats DN. Additionally, it proposes a novel approach for identifying the active components of traditional Chinese medicine.

## 1. Introduction

Diabetes mellitus (DM) is regarded as one of the most predominant and critical chronic disorders today, resulting in severe, disabling, and expensive complications, as well as decreasing lifespan [1]. Currently, the worldwide incidence of DM exceeds 10% [1]. Among these individuals, approximately 30%–40% will experience diabetic nephropathy (DN) at some point in their lives [2]. DN, a frequent microvascular complication of Type 1 diabetes (T1D) and Type 2 diabetes (T2D), stands as the primary cause of chronic and end‐stage renal disorders [3]. The development of DN is intricate and comprises several pathological mechanisms, including inflammation, oxidative stress, and epithelial–mesenchymal transition [4]. Recent investigations have highlighted the pivotal function of inflammatory pathways in the progression of DN [5]. Despite the advent of treatments like sodium–glucose cotransporter 2 inhibitors, which diminish inflammation and oxidative stress and show efficacy in lowering the risk of main adverse events and slowing renal disease progression in T2D patients, overall therapeutic outcomes remain suboptimal [4, 6]. Thus, gaining deeper insights into the pathogenesis of DN and identifying novel therapeutic agents is of significant clinical importance.

Traditional Chinese medicine (TCM), a traditional healing practice in Asia and a form of complementary and alternative medicine in Western healthcare, has garnered significant attention in the life sciences [7]. Natural products obtained from TCM have been widely investigated as potential treatments for various chronic conditions [8]. Astragaloside IV (AS‐IV), the primary active element in *Astragalus*, displays multiple pharmacological actions, including anti‐inflammatory, antifibrotic, antidiabetic, and immune‐regulating properties through numerous signaling pathways [9]. Current investigation and clinical observations illustrate that AS‐IV holds substantial promise for applications in DN. For instance, it improved renal function and reduced podocyte damage in db/db mice by mitigating NLRP3 inflammasome‐mediated inflammation, thereby slowing the progression of DN [10]. Additionally, AS‐IV suppressed renal mesangial hyperproliferation and renal fibrosis by the TGF‐*β*1/Smad/miR‐192 pathway [11]. Despite its potential, the protective mechanisms of AS‐IV in DN are not completely comprehended. Hence, a deeper understanding of the molecular AS‐IV mechanisms could offer novel insights into its therapeutic potential.

In order to better understand disease mechanisms and drug actions, a new area called network pharmacology is emerging. This field integrates bioinformatics with pharmacology [12]. Given the complex composition of TCM, with its multiple targets and extensive signaling pathways, studying it in detail poses significant challenges [13]. As a result, an increasing number of studies are employing network pharmacology techniques to offer more foundational and scientific explanations for TCM‐related research [13, 14]. In summary, this research seeks to evaluate the molecular mechanisms by which AS‐IV treats DN by leveraging multiple databases to pinpoint potential AS‐IV drug targets and DN disease targets, culminating in the identification of combined AS‐IV‐DN targets. Protein–protein interaction (PPI) networks were developed to examine the correlations between these targets. Subsequently, Kyoto Encyclopedia of Genes and Genomes (KEGG) and Gene Ontology (GO) analyses were conducted to clarify the biological roles of the critical genes. Lastly, in vivo trials were conducted to confirm the validity of these essential targets.

Our study identified the core targets of AS‐IV for DN treatment, including MMP‐9, myeloperoxidase (MPO), IL‐6, and IL‐1*β*, revealing that AS‐IV may slow the DN progression by suppressing the inflammatory response via inhibition of the TNF‐*α* pathway. We present a reliable approach to the study of TCM, which suggests the promise of a new clinical treatment for DN.

## 2. Materials and Methods

### 2.1. Animal Grouping and Treatment

Six‐week‐old male db/db (C57BLKS/J‐leprdb/leprdb) and db/m mice were obtained from Gempharmatech Co. Ltd. (Jiangsu, China). The investigation received approval from the Ethics Committee of Medical Science Research, Affiliated Hospital of Jining Medical University (Approval No.: 2024‐03‐B003), and complied with NIH protocol for the Care and Use of Laboratory Animals. Mice were kept under a 12‐h light/dark cycle with unrestricted food and water. After 2 weeks of acclimatization in a specific pathogen‐free environment, they were classified into three groups in a random manner (*n* = 10 each): normal control (Con, db/m), DN (db/db), and AS‐IV‐treated DN (db/db + AS‐IV). Db/db mice possess a mutation resulting in the deletion of the leptin receptor, rendering them prevalent models for investigating T2D. The inherent genetic makeup of db/db mice predisposes them to obesity and associated diabetic complications, including nephropathy [15]. Prior to AS‐IV treatment, the DN model in db/db mice was strictly validated using two core criteria to confirm successful induction: (1) fasting blood glucose (FBG) levels ≥ 16.7 mmol/L (measured via Accu‐Chek Active blood glucose meters, Roche Diagnostic, Switzerland) after a 12‐h fast, indicating persistent hyperglycemia; (2) 24‐h urinary albumin excretion (UAE) significantly higher than that of db/m control mice (quantified via immunoturbidimetric assay using metabolic cages), reflecting early glomerular filtration barrier impairment. Only db/db mice meeting both criteria were included in the DN group [15]. In the AS‐IV group, mice received daily gavage of 20 mg/kg body weight (BW) AS‐IV (Nanjing Spring & Autumn Biological Engineering Co. Ltd., Nanjing, China) for 10 weeks postmodel confirmation [16]. The Con and DN groups received equal volumes of saline. At the experimental endpoint, mice were euthanized under deep anesthesia with isoflurane (RWD Life Science Co. Ltd., Shenzhen, China). After loss of pedal reflex, an overdose of sodium pentobarbital (Sigma‐Aldrich, St. Louis, MO, United States) (150 mg/kg, intraperitoneal) was administered to achieve euthanasia. Death was confirmed by the absence of heartbeat and respiration.

### 2.2. Biochemical Indicator Measurement

FBG levels were assessed using Accu‐Chek Active blood glucose meters (Roche Diagnostic, Switzerland). Urine collection over 24 h was conducted via metabolic cages, and UAE was quantified via an immunoturbidimetric assay. BW was recorded for each group of mice. Serum creatinine (Scr) and blood urea nitrogen (BUN) concentrations were analyzed from orbital blood samples.

### 2.3. Histopathological Evaluation

The kidney tissues underwent preservation in 4% paraformaldehyde, dehydration in ethanol, paraffin embedding, and sectioning. To assess the histological alterations, hematoxylin–eosin (HE), Masson′s trichrome (MASSON), and periodic acid–Schiff (PAS) were utilized to stain the sections.

### 2.4. Gene Expression Omnibus (GEO) Data Retrieval and Preprocessing

The GEO database was the source of microarray data of peripheral blood mononuclear cells (PBMCs) from DN individuals (https://www.ncbi.nlm.nih.gov/). The GSE142153 dataset comprised 10 healthy controls and 23 DN samples. Raw data were subjected to quality control, weighted gene coexpression network analysis (WGCNA), and differential gene expression analysis to uncover pivotal biological processes (BPs) and pathways in DN pathogenesis.

### 2.5. Key Module Identification and WGCNA

WGCNA is extensively applied in diverse systems biology analyses by creating scale‐free networks that link gene expression levels with clinical traits. To ensure robust network construction, samples were first normalized and outlier samples excluded. The selection of soft‐threshold power depended on the criteria of a scale‐free network, and genes in the top 25% variance were computed using a power function. The soft‐thresholding power (*β*) was systematically selected as 6 through evaluating the scale‐free topology fitting index (*R* ^2^) and mean connectivity. This choice was validated by the achievement of an *R* ^2^ value of 0.87, which meets and exceeds the recommended threshold of *R* ^2^ ≥ 0.85 for approximate scale‐free topology, thereby ensuring the coexpression network adheres to the core characteristics of scale‐free networks. Genes in the top 25% variance were computed using a power function. We next estimated the dissimilarity (1‐topological overlap matrix [TOM]) by transforming the adjacency matrix into a TOM. Genes were hierarchically clustered, and modules were defined via the dynamic tree cut method (deepSplit = 2, minimum size = 30). To reduce redundancy among highly similar modules and improve the interpretability of biologically meaningful modules, a height cutoff of 0.25 was strictly applied for module merging. Extremely identical modules were combined (height cutoff = 0.25) using the WGCNA module‐saving function. Pearson′s correlation tests evaluated associations between clinical traits and modules, identifying significant ones. Module membership (MM) was identified as the relationship between gene profiles and module eigengenes (MEs), and gene significance (GS) as the absolute relationship between external traits and gene profiles. Genes with the uppermost MM and GS values in the target module were further analyzed.

### 2.6. Extraction of AS‐IV Targets

Potential AS‐IV targets were identified via the CTD database (http://ctdbase.org/), SwissTargetPrediction database (http://swisstargetprediction.ch/), Target Prediction database (https://prediction.charite.de/index.php), and TargetNet database (http://targetnet.scbdd.com/home/index/).

### 2.7. Extraction of DN Targets

Potential targets of DN were identified using GeneCards (https://www.genecards.org/), DigSee (http://210.107.182.61/geneSearch/), and DisGeNET (https://www.disgenet.org/home/).

### 2.8. Mapping Interaction Molecule

Potential AS‐IV targets and molecules associated with DN were mapped and analyzed with the aim of identifying molecules that may be targets of AS‐IV action for DN treatment.

### 2.9. Constructing Protein Interaction Network

The STRING database (https://cn.string-db.org/), along with Cytoscape software (v3.9.1), was employed to create a PPI network atlas. In this network, nodes symbolize individual proteins, while edges denote interactions between these proteins. Topological characteristics of the PPI network were evaluated via Cytoscape′s Network Analyzer tool, emphasizing the “degree” of each node. Subsequently, the MCODE plug‐in was utilized to identify a core cluster within the PPI network.

### 2.10. Functional Enrichment Analysis

We conducted the GO and KEGG analyses of core targets via the R language software toolkit, with bubble plots provided for visualization. To control the false discovery rate (FDR) during multiple testing, the Benjamini–Hochberg (BH) method was used for *p* value adjustment. GO analysis screened for BPs, molecular functions (MFs), and cellular components (CCs) [17]. KEGG analysis determined the main signaling pathways participating in BP. A *p*.adjust < 0.05 (after BH adjustment) was set as the threshold for statistically significant enrichment.

### 2.11. Differential Gene Analysis and Receiver Operating Characteristic (ROC) Curve Analysis

To enhance the reliability of the data, the potential target molecules for AS‐IV treatment of DN obtained from online databases were cross‐referenced with the differentially expressed molecules identified by GEO analysis. Through in‐depth analysis using the R language programming package, not only was the differential expression of these molecules in the GEO dataset explored but also their ROC curves were analyzed to assess their diagnostic efficacy as potential biomarkers.

### 2.12. Western Blot Analysis

According to our previous study [18], protein expression levels of MMP‐9, MPO, IL‐6, IL‐1*β*, and TNF‐*α* in renal tissues of different groups were detected by western blot analysis. Detailed information about the antibodies is provided below: MMP‐9 (YT1892, Immunoway, Jiangsu, China), MPO (YM4746, Immunoway, Jiangsu, China), IL‐6 (#32064, Signalway, MA, United States), IL‐1*β* (#41059, Signalway, MA, United States), TNF‐*α* (YT4689, Immunoway, Jiangsu, China), and *β*‐actin (#21800, Signalway, MA, United States). The ECL Chemiluminescence Kit (Beyotime, China) was used to observe chemiluminescence signals. ImageJ software (National Institutes of Health, Bethesda, MD, United States) was used to quantify the blots. The quantification of protein bands was performed using relative fold change. Band intensities were normalized to *β*‐actin as the loading control, and the relative expression levels were calculated by comparing with the control group. All western blot experiments were performed in triplicate to ensure reproducibility and reliability of the results.

### 2.13. Statistical Analysis

GraphPad Prism v5.0.0 was utilized to conduct statistical analyses (GraphPad Software, United States). Data are described as mean ± standard deviation (SD). The *t*‐test was employed for group comparisons, with *p* < 0.05 considered significant.

## 3. Results

### 3.1. AS‐IV Effects on Metabolic Parameters and Kidney Histopathology in DN Mice

Compared to the Con group, the DN group displayed a significant elevation in levels of FBG, BW, UAE, Scr, and BUN. However, compared to the DN group (*p* <0.05), these concentrations were noticeably decreased in the AS‐IV group (*p* <0.05) (Figures 1a, 1b, 1c, 1d, and 1e). HE staining illustrated extensive glomerular hypertrophy (red arrows) and vacuolar degradation in renal tubular epithelial cells (black arrows) in the DN group compared to the Con group. PAS staining illustrated notable glomerular hypertrophy (red arrows) and mesangial matrix expansion (black arrows) in the DN group in comparison to the Con group. In addition, high interstitial fibrosis (black arrows) was observed in the DN group by MASSON staining. However, AS‐IV treatment significantly improved renal histopathologic changes in DN mice (Figure 1f).

Figure 1AS‐IV impacts on metabolic parameters and renal histopathology in DN mice. (a) Fasting blood glucose, (b) body weight, (c) urinary albumin excretion, (d) serum creatinine, and (e) blood urea nitrogen were assessed. (f) Renal histopathological alterations were assessed by HE, PAS, and Masson staining (scale bar = 20 * μ*m).  ^∗^ *p* < 0.05,  ^∗∗^ 0.01, and  ^∗∗∗^ 0.001 versus Con; ^#^ *p* < 0.05, ^##^ 0.01, and ^###^ 0.001 versus DN.

**Figure:**
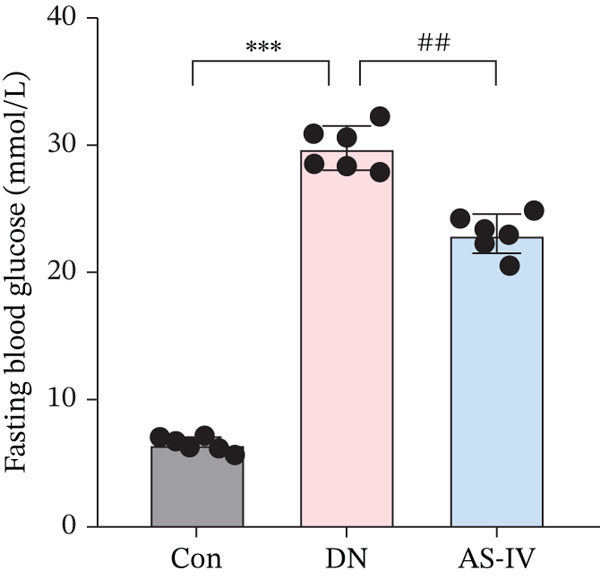
(a)

**Figure:**
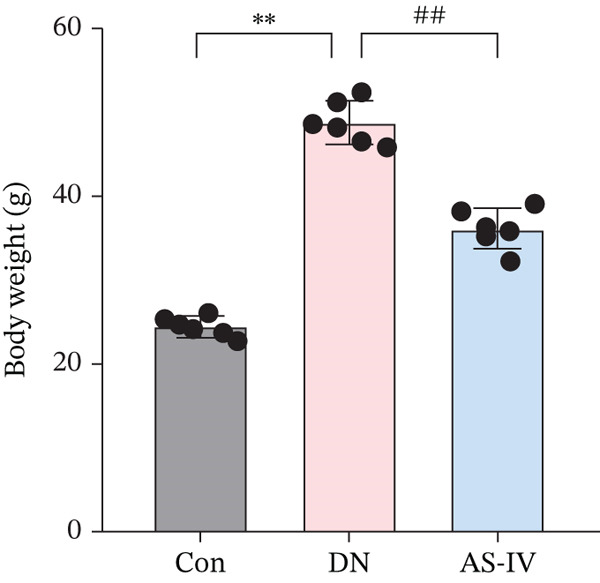
(b)

**Figure:**
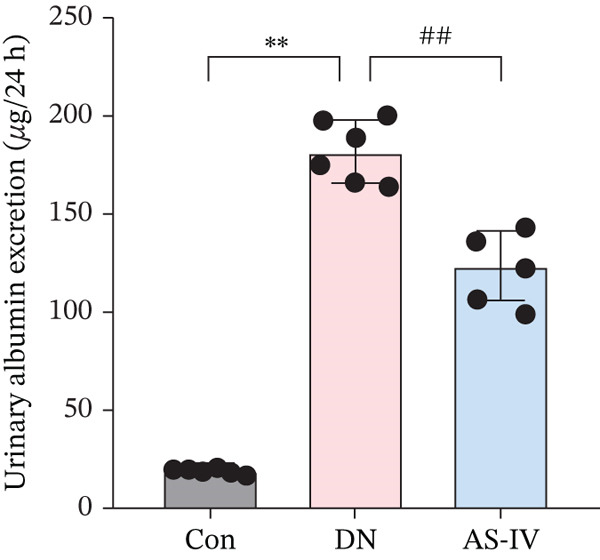
(c)

**Figure:**
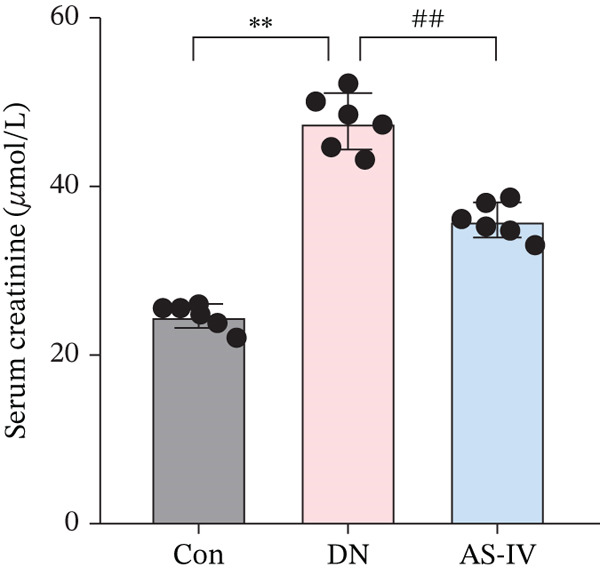
(d)

**Figure:**
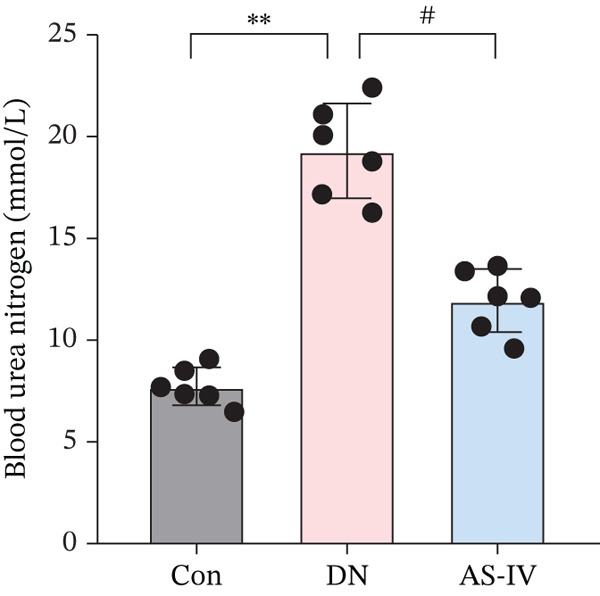
(e)

**Figure:**
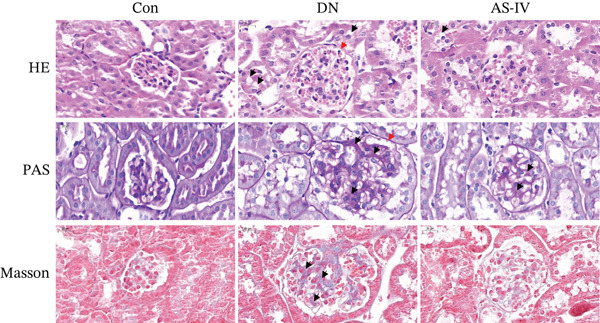
(f)

### 3.2. Identification of Differentially Expressed Genes in DN

Following the established screening criteria, we retrieved and selected the GSE142153 dataset from the GEO database for this study. This dataset includes 10 healthy Con samples and 23 DN patient samples, intended to identify gene expression differences under DN conditions. Through rigorous screening, 424 significantly differentially expressed genes were detected, with 171 genes overexpressed and 253 genes suppressed (Figure 2a).

Figure 2GSE142153 bioinformatics analysis. (a) The volcano map analysis of normal and DN patients. (b) Construction of network module clustering map and feature module heatmap for GSE142153 coexpression using WGCNA. (c) Network heatmap of WGCNA coexpression modules. Gradual saturation in red indicates a high degree of overlap between functional modules. (d) GO/KEGG functional analysis. (e) GSEA.

**Figure:**
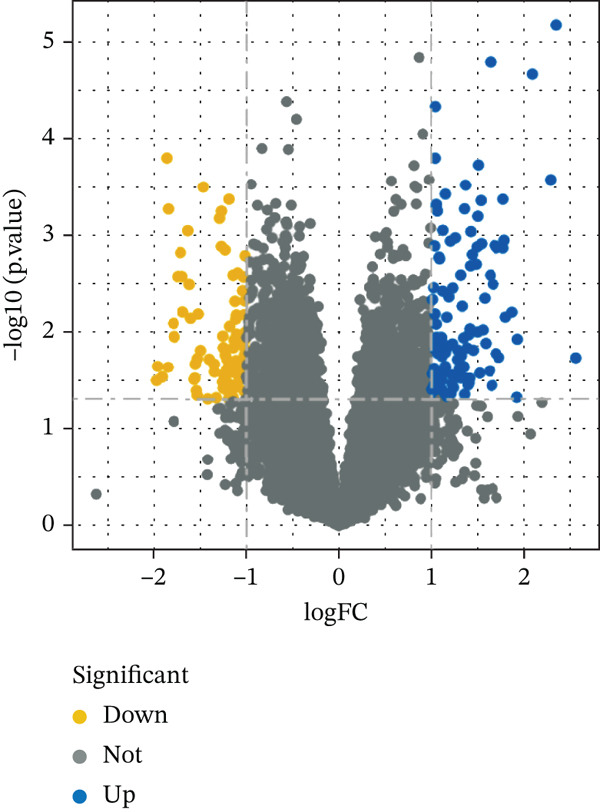
(a)

**Figure:**
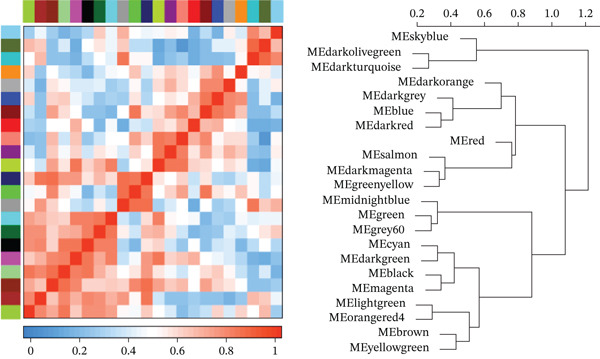
(b)

**Figure:**
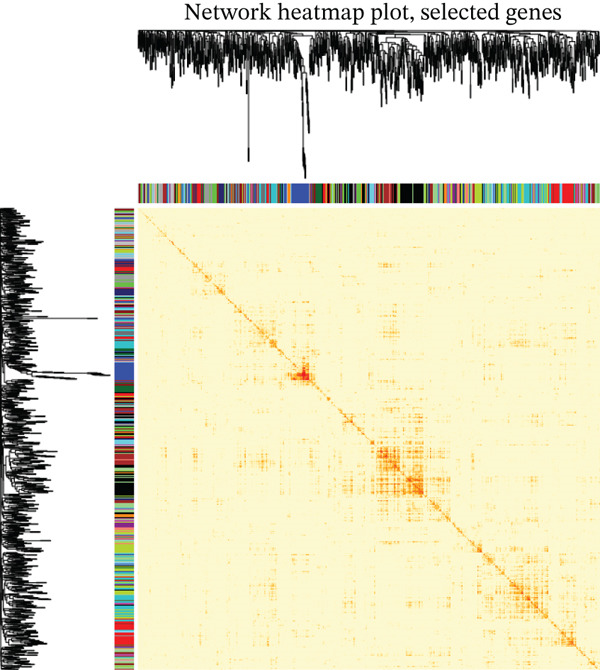
(c)

**Figure:**
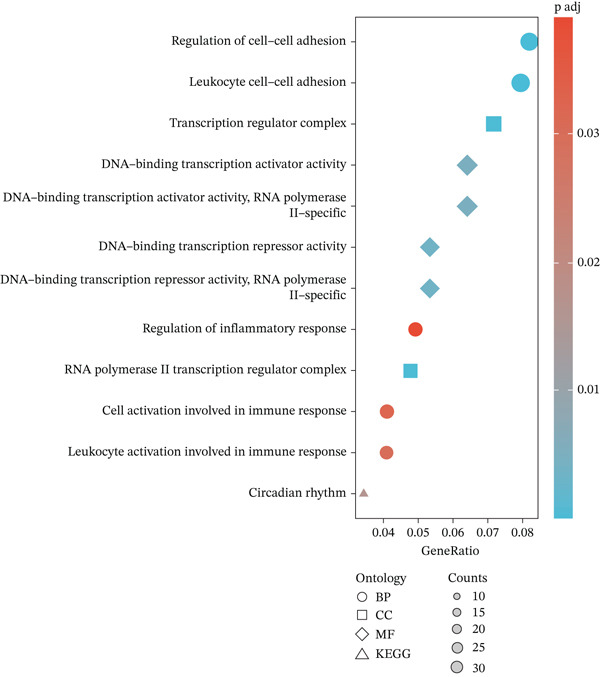
(d)

**Figure:**
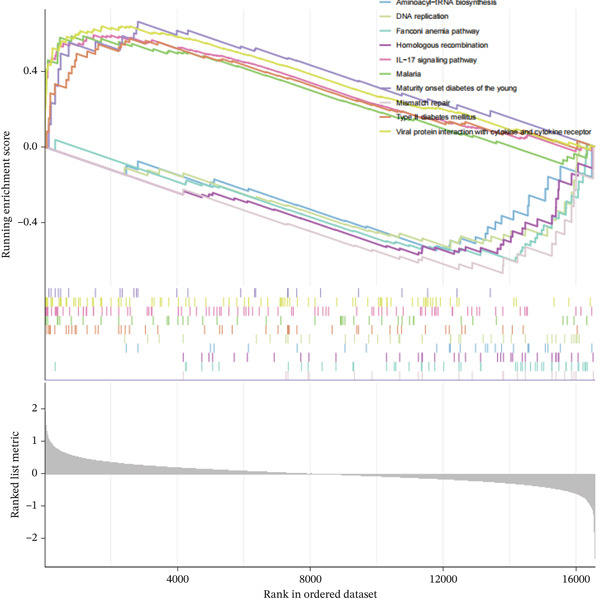
(e)

### 3.3. Identification of DN‐Related Gene Modules

Using the WGCNA package, a coexpression network was created depending on the standardized gene expression data from the GSE142153 dataset. Module–trait relationship analysis indicated several modules associated with DN, integrating DN into the clustering tree and generating a unified Mes correlation heatmap. The overlap in the heatmap suggested a certain degree of correlation between different modules (Figure 2b). WGCNA constructed 22 coexpression modules, among which the yellow–green module (MEyellowgreen) showed the strongest correlation with DN phenotypes (*r* = 0.68, *p* = 0.03) and the most significant negative correlation with normal controls (*r* = −0.68, *p* = 0.03) (Supporting Information 1: Figure S1). The gene correlation heatmap was generated based on the TOM (Figure 2c). The yellow–green module exhibited the most significant differences and underwent further functional analysis. Results included processes such as leukocyte cell–cell adhesion, regulation of inflammatory response and leukocyte adhesion, transcription regulator complex, DNA‐binding transcription activator and repressor activities (specific to RNA polymerase II), circadian rhythm, RNA polymerase II transcription regulator complex, and cell and leukocyte stimulation involved in immune response (Figure 2d). Additionally, GSEA identified five significantly upregulated and five significantly downregulated pathways, including aminoacyl‐tRNA biosynthesis, DNA replication, the Fanconi anemia pathway, homologous recombination, the IL‐17 pathway, malaria, maturity‐onset DM of the young, mismatch repair, T2D, and viral protein interaction with cytokine receptor and cytokine (Figure 2e).

### 3.4. Acquisition of AS‐IV‐Related Targets

The molecular configuration of AS‐IV was acquired from the PubChem database (Figure 3a,b). Corresponding potential targets were identified through the CTD, SwissTargetPrediction, TargetNet, and Target Prediction databases. Data normalization via UniProt identified 41, 21, 54, and 97 target molecules, respectively. Integration and deduplication of these datasets yielded a final set of 192 potential AS‐IV target molecules.

Figure 3Network pharmacology analysis. (a) 2D molecular structure of AS‐IV. (b) 3D molecular structure of AS‐IV. (c) AS‐IV and DN Wayne mapping analysis. (d) PPI network built using the STRING online database. (e) Use Cytoscape′s built‐in plugins to analyze nodes in a PPI network and analyze their importance depending on node “degree.” With increasing node degree, associated biological functions in the network increase, with higher degrees represented by darker colors and larger shapes.

**Figure:**
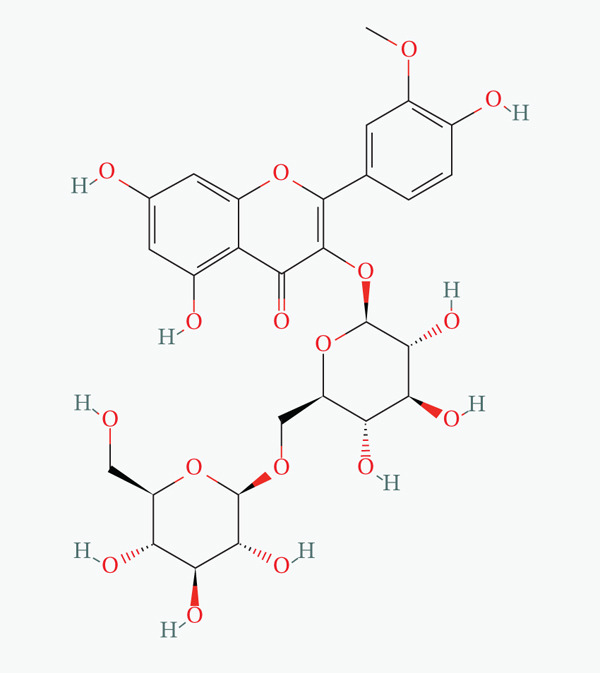
(a)

**Figure:**
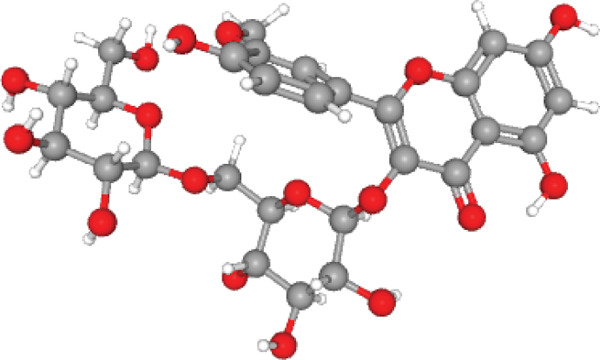
(b)

**Figure:**
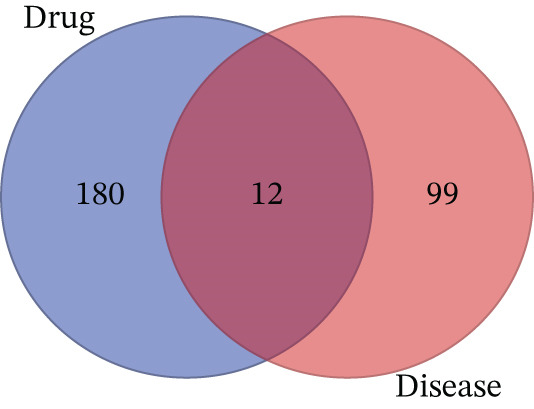
(c)

**Figure:**
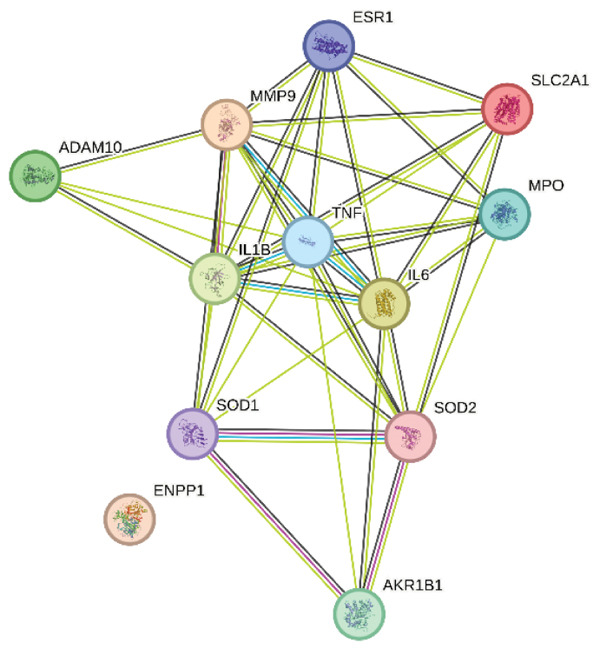
(d)

**Figure:**
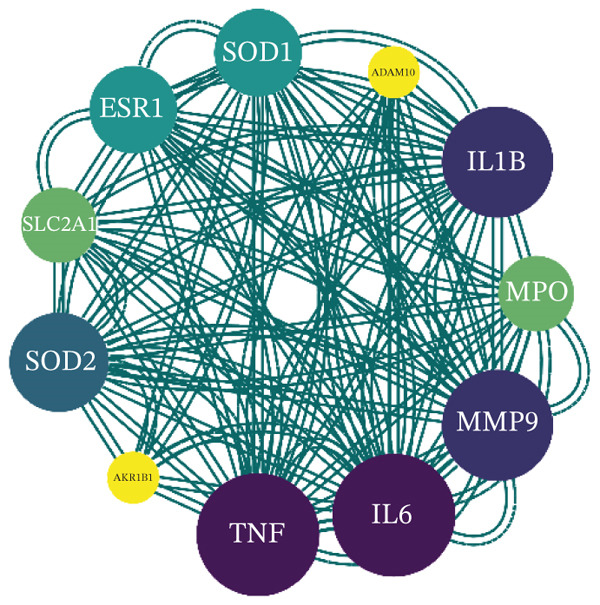
(e)

### 3.5. Acquisition of DN‐Correlated Targets

DN‐related disease molecules were screened from the GeneCards, DigSee, and DisGeNET databases. After data normalization, 4050, 1189, and 1226 candidate disease molecules were confirmed from these databases, respectively. Intersection analysis of these datasets identified 111 common disease‐related molecules.

### 3.6. Mapping of Drug–Disease Target

A Venn diagram analysis of the 192 potential AS‐IV targets and the 111 DN‐related pathological molecules identified 12 key targets potentially mediating drug–disease interactions (Figure 3c).

### 3.7. PPI Network Analysis

Utilizing the STRING database, a PPI network encompassing the 12 drug–disease interaction targets was developed and thoroughly examined. This network comprised 12 nodes and 40 edges, with an average node connectivity of 6.67 and a PPI network significance *p* value < 0.01 (Figure 3d). Subsequently, the STRING‐constructed PPI network was introduced into Cytoscape for additional processing. Through various Cytoscape plugins, statistical analysis of network nodes was performed, and node importance was assessed based on connectivity. Nodes with higher connectivity demonstrated more significant biological functions and were marked with darker colors in Figure 3e for distinction.

### 3.8. GO Analysis

GO analysis of the 12 key targets was performed via R software, and the results were visualized using bioinformatics tools. In the BP class, 1103 terms were observed, with the Top 5 of significant enrichment (*p*.adjust < 0.05) primarily related to cellular responses to chemical stress, oxidative stress, and reactive oxygen species (ROS), as well as regulating inflammatory response and cellular response to oxidative stress (Figure 4a). In the MF class, the Top 5 significantly enriched MF terms (*p*.adjust < 0.05) included cytokine receptor binding, receptor ligand, signaling receptor activator, cytokine, and antioxidant activities (Figure 4b).

Figure 4Biological function analysis. GO enrichment analysis, comprising the (a) biological processes (BPs) and (b) molecular function (MF) items. (c) KEGG pathway analysis. Node colors are shown in a red‐to‐blue gradient in order of *p* value from largest to smallest, and node sizes are in ascending order of gene number.

**Figure:**
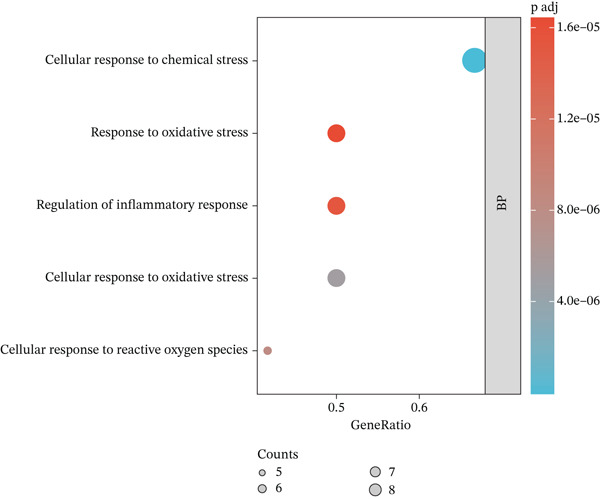
(a)

**Figure:**
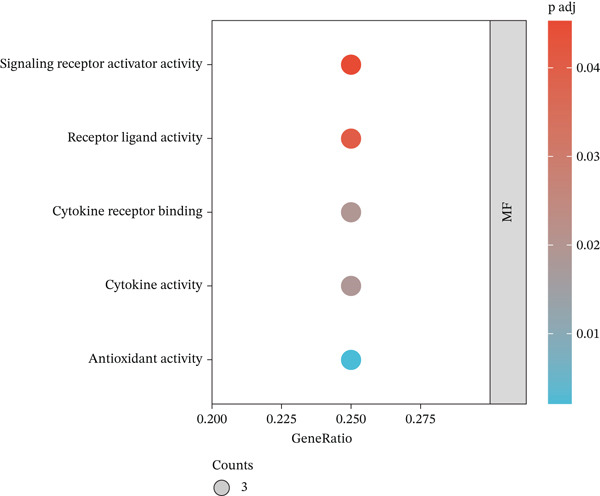
(b)

**Figure:**
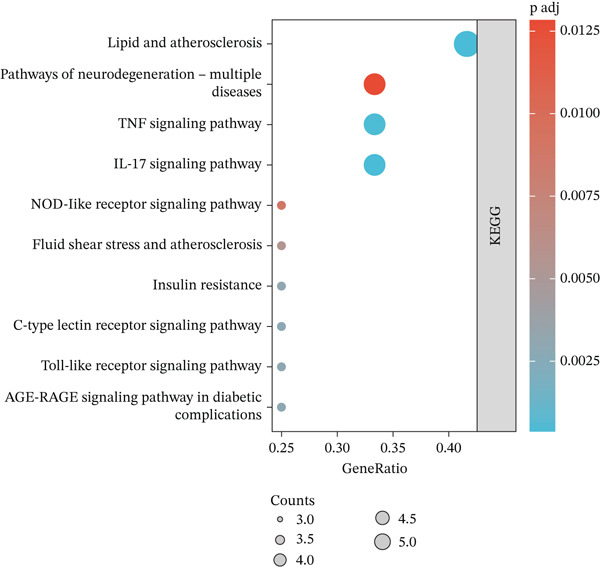
(c)

### 3.9. KEGG Enrichment Analysis

KEGG analysis of the 12 core targets using R and Cytoscape′s ClueGO plugin identified 54 significant pathways (*p* < 0.01). Comprehensive evaluation highlighted 10 key pathways: lipid and atherosclerosis, neurodegeneration (multiple diseases), fluid shear stress and atherosclerosis, insulin resistance, TNF, IL‐17, NOD‐like receptor, C‐type lectin receptor, Toll‐like receptor, and AGE‐RAGE signaling pathways in DM complications (Figure 4c).

### 3.10. Key Cluster Gene Validation

To enhance the reliability of our findings, key genes identified through network pharmacology were validated for expression differences between normal and DN groups using the GEO database. As shown in Figure 5a, IL‐1*β*, IL‐6, MMP‐9, and MPO exhibited statistically significant expression alterations in DN patients compared to healthy controls. To further evaluate their potential as diagnostic biomarkers for DN, we performed ROC curve analysis. The corresponding ROC curves are presented in Figure 5b, and detailed quantitative results are provided in Supporting Information 2: Table S1.

Figure 5Differential expression and diagnostic value of core genes in GEO sequencing data. (a) Key cluster boxplot ( ^∗^ *p* < 0.05 and  ^∗∗^ 0.01; # indicates queshi3; ns, not significant). (b) ROC curves for MMP‐9, MPO, IL‐6, and IL‐1*β*.

**Figure:**
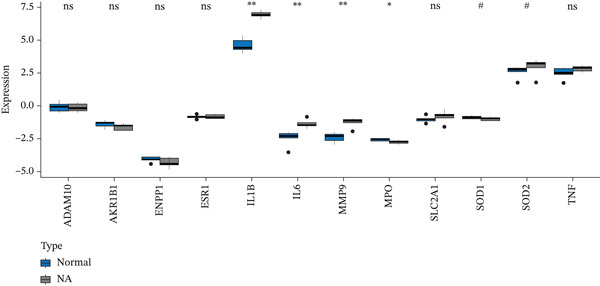
(a)

**Figure:**
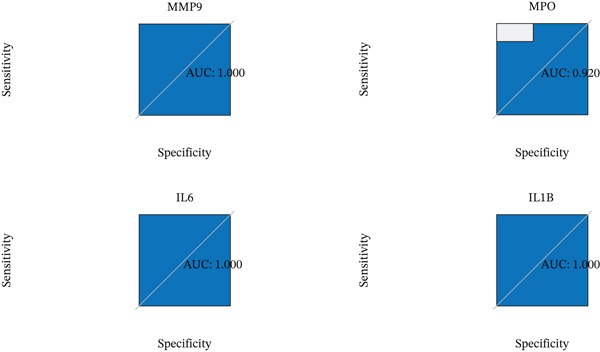
(b)

### 3.11. Key Target Gene Expression in DN Mice After AS‐IV Treatment

Western blotting was utilized to estimate alterations in protein expression of the key targets observed in DN mice treated with AS‐IV. Compared to the Con group, the expression of MMP‐9, MPO, and IL‐6/1*β* was significantly raised in the DN group (*p* < 0.05), but these alterations were reversed following AS‐IV treatment (*p* < 0.05) (Figure 6). These outcomes indicate that MMP‐9, MPO, and IL‐6/1*β* may be pivotal in mediating AS‐IV therapeutic effects on DN.

Figure 6Expression alterations of four core targets in Con, DN, and AS‐IV groups. (a) Representative protein bands of MMP‐9, MPO, IL‐6, and IL‐1*β*. (b–e) Quantification of respective protein levels.  ^∗^ *p* < 0.05,  ^∗∗^ 0.01, and  ^∗∗∗^ 0.001 versus Con; ^#^ *p* < 0.05, ^##^ 0.01, and ^###^ 0.001 versus DN.

**Figure:**
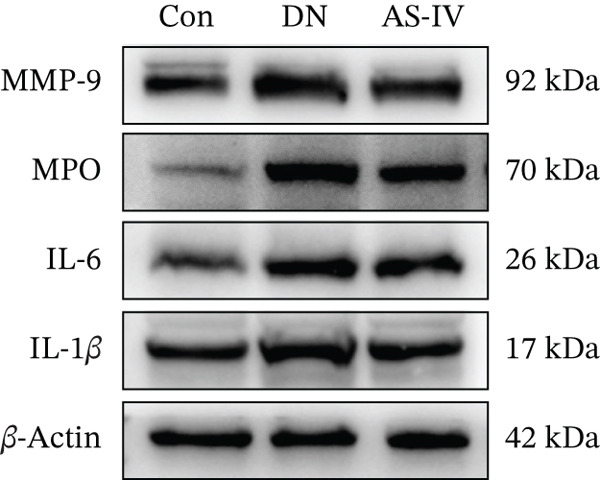
(a)

**Figure:**
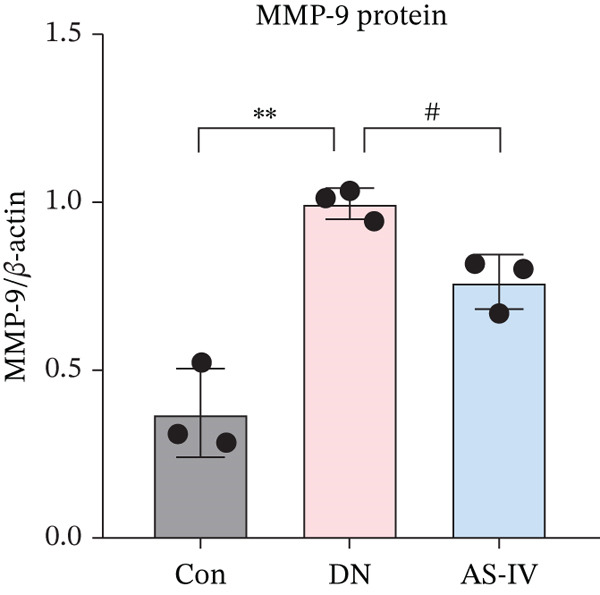
(b)

**Figure:**
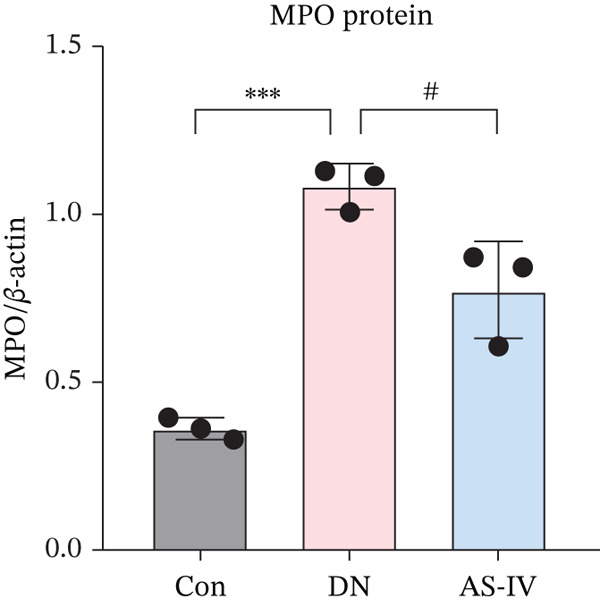
(c)

**Figure:**
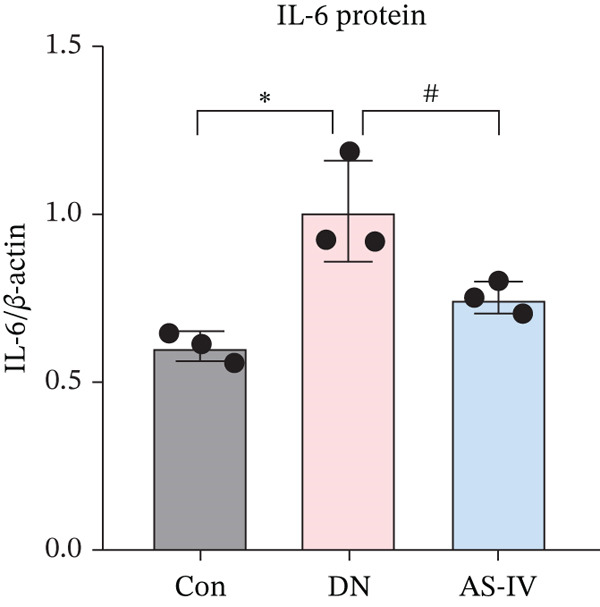
(d)

**Figure:**
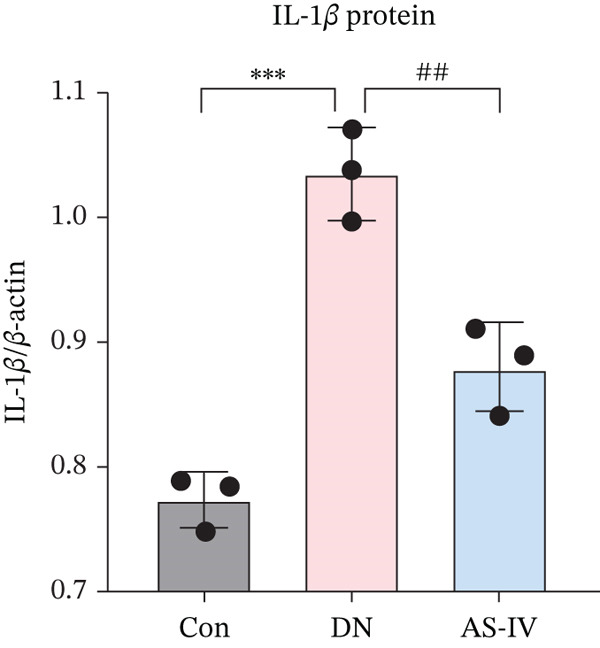
(e)

### 3.12. AS‐IV Treatment Might Improve Inflammatory Response in DN Mice by Inhibiting the TNF‐*α* Pathway

The TNF pathway is essential in the development and pathogenesis of DN [19]. To delve deeper into the AS‐IV therapeutic mechanism in DN, we assessed TNF‐*α* protein levels. Western blotting outcomes illustrated that, compared to the Con group, TNF‐*α* was considerably overexpressed in the DN group (*p* < 0.05). Conversely, TNF‐*α* concentrations were significantly reduced in the AS‐IV group relative to the DN group (*p* < 0.05) (Figure 7).

Figure 7TNF‐*α* signaling in AS‐IV‐mediated therapeutic effects on DN. (a) Representative protein bands of TNF‐*α* in Con, DN, and AS‐IV groups. (b) Quantification of TNF‐*α* protein levels among groups.  ^∗^ *p* < 0.05,  ^∗∗^ 0.01, and  ^∗∗∗^ 0.001 versus Con; ^#^ *p* < 0.05, ^##^ 0.01, and ^###^ 0.001 versus DN.

**Figure:**
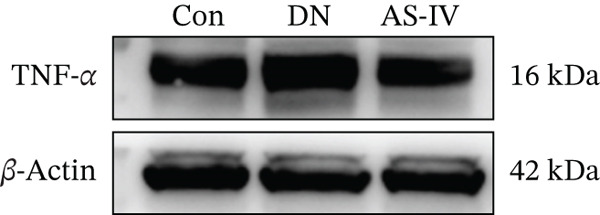
(a)

**Figure:**
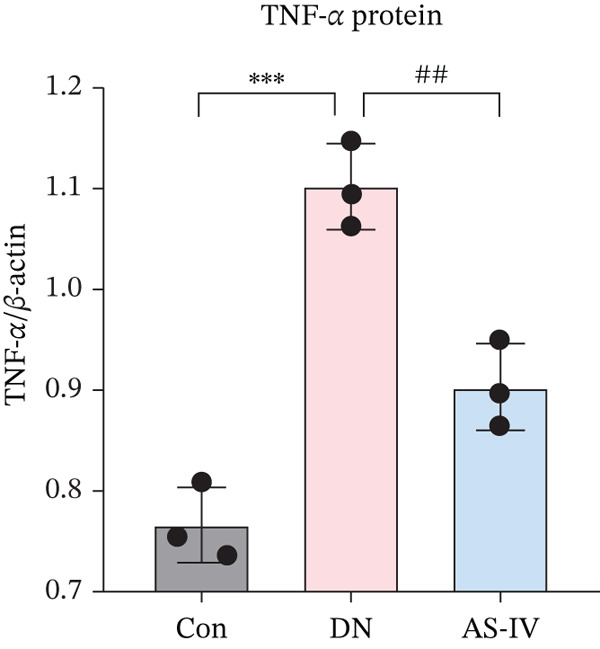
(b)

## 4. Discussion

DN represents a significant microvascular complication of DM, characterized by persistent hyperglycemia‐induced renal dysfunction and progressive renal failure [20]. Its pathogenesis involves intricate networks of inflammation, oxidative stress, and epithelial–mesenchymal transition [21]. Current clinical treatments centered on glycemic and blood pressure control have limited efficacy in halting DN progression [21], highlighting the need for novel therapeutic strategies. TCM has gained attention for its multitarget and multipathway advantages in chronic disease management, making it a promising resource for DN therapy [22].

AS‐IV (C_41_H_68_O_14_, molecular weight = 784.97) is a principal active component of *Astragalus*, with well‐documented anti‐inflammatory, antifibrotic, and antidiabetic properties [23, 24]. Accumulating evidence supports its renoprotective potential in DN: Prior studies have reported its efficacy via isolated pathways, including NLRP3 inflammasome inhibition [10], TGF‐*β*1/Smad/miR‐192 modulation [11], eNOS activation [25], and Wnt/*β*‐catenin suppression [24]. Consistent with these findings, our study demonstrated that AS‐IV reduces key metabolic parameters (FBG, BUN, Scr, and UAE) and alleviates renal histopathological lesions in DN mice. Notably, UAE—an indicator of renal impairment—was significantly elevated in the DN group and markedly reduced by AS‐IV treatment, aligning with improvements in other renal function markers and histopathology, further validating AS‐IV′s therapeutic promise for DN.

To investigate the potential mechanisms of AS‐IV in DN treatment, our study utilized a network pharmacology strategy to predict AS‐IV‐related and DN‐related targets, identifying 12 potential intersection targets. Prior to experimental validation, we analyzed their expression profiles in the GEO database: Figure 5a illustrated statistically significant changes in the expression of IL‐1*β*, IL‐6, MMP‐9, and MPO between DN and normal groups. Additionally, ROC analysis (Figure 5b) confirmed these four targets as highly specific and sensitive biomarkers for DN. These targets are not only hub nodes in the predicted regulatory network but have also been extensively reported to be closely associated with DN‐related inflammation, oxidative stress, and renal fibrosis [26–31]. Based on these multidimensional evidences, we prioritized these four targets for Western blot validation. The results confirmed AS‐IV′s regulatory effect on their protein expression, directly supporting the network pharmacology hypothesis that AS‐IV exerts renoprotection via multitarget regulation.

MMP‐9, a member of the matrix metalloproteinase family, maintains extracellular matrix homeostasis critical for glomerular integrity. Elevated MMP‐9 levels are linked to DM and renal disease progression, with increased serum and urinary concentrations in DN patients and animal models [27, 28]. MPO, a leukocyte‐derived heme protein, mediates vascular damage via ROS production, and elevated MPO levels are associated with T2DM and nephropathy [29–31]. Our study confirmed significantly increased MMP‐9 and MPO expression in the DN group, with AS‐IV downregulating these proteins—highlighting a novel regulatory role of AS‐IV in extracellular matrix remodeling and oxidative stress in DN.

Inflammatory responses are closely linked to DN development, with interleukins playing pivotal roles [32, 33]. IL‐6 and IL‐1*β* are key inflammatory cytokines implicated in DN pathogenesis: Clinical studies report higher IL‐6 levels in DN patients, with gene polymorphisms increasing disease risk [34, 35], while IL‐1*β* levels correlate with DN severity and guide early diagnosis [36–38]. Consistent with previous reports, our study showed elevated IL‐6/1*β* levels in the DN group, with AS‐IV significantly reducing these levels—reinforcing AS‐IV′s anti‐inflammatory potential in DN.

To explore AS‐IV′s mechanism in DN, we performed GO and KEGG enrichment analyses. GO analysis linked AS‐IV to cellular responses to chemical/oxidative stress, inflammatory regulation, and ROS signaling. KEGG enrichment identified TNF, IL‐17, and AGE‐RAGE pathways (key in DM complications), pointing to AS‐IV′s protective role via TNF signaling. Notably, TNF‐*α* was not among the initial 12 overlapping AS‐IV‐DN targets but was the central effector of the KEGG‐enriched TNF pathway (*p* < 0.01) and a well‐documented mediator of DN‐related inflammation, oxidative stress, and renal damage [19]. We thus selected TNF‐*α* for validation. Consistent with its pivotal role in DN pathogenesis [38], TNF‐*α* was upregulated in DN mice, and AS‐IV reversed this elevation—confirming AS‐IV′s anti‐inflammatory effects via TNF‐*α* inhibition, complementing KEGG results, and establishing a complete regulatory network linking AS‐IV, core targets (MMP‐9, MPO, IL‐6, and IL‐1*β*), and the TNF pathway in DN. This integrated regulatory network, identified via combined network pharmacology and experimental validation, represents the most novel finding of our study, providing a comprehensive mechanistic explanation for AS‐IV′s renoprotection in DN beyond isolated pathway reports.

## 5. Conclusion

This investigation, integrating network pharmacology and in vivo experimental validation, identifies AS‐IV′s core targets in DN treatment, revealing its therapeutic mechanism through TNF‐*α* pathway inhibition. These outcomes offer a robust theoretical and experimental foundation for AS‐IV clinical application in treating DN.

## Author Contributions

N.W. and L.L. conceptualized the study. H.L. and B.W. designed the study, conducted the experiments, and analyzed the data. H.C., Y.L., and Z.S. assisted with experiments and analysis. Y.C. and X.L. evaluated the results. H.L. and B.W. drafted the manuscript. H.L. and B.W. made equal contributions to this investigation H.L. and B.W. contributed equally to this work.

## Funding

This study was funded by the Research Fund for Academician Lin He New Medicine (JYHL2022FMS02) and the Shandong Province Traditional Chinese Medicine Science & Technology Project (M‐2023062).

## Disclosure

All authors reviewed and gave their approval to the final manuscript.

## Ethics Statement

The Ethics Committee of Medical Science Research, Affiliated Hospital of Jining Medical University (Approval No.: 2024‐03‐B003), approved the study protocol, and the study strictly adheres to the ARRIVE reporting guidelines to ensure the reliability and transparency of experimental design and results.

## Consent

The authors have nothing to report.

## Conflicts of Interest

The authors declare no conflicts of interest.

## Supporting Information

Additional supporting information can be found online in the Supporting Information section.

## Supporting information


**Supporting Information 1** Figure S1: WGCNA coexpression modules.

**Supporting Information 2** Table S1: ROC curve quantitative data of core targets.

## Data Availability

The data and materials underpinning the results of this study are provided within this article and its supporting information.

## References

[1] Sun H. , Saeedi P. , Karuranga S. , Pinkepank M. , Ogurtsova K. , Duncan B. B. , Stein C. , Basit A. , Chan J. C. N. , Mbanya J. C. , Pavkov M. E. , Ramachandaran A. , Wild S. H. , James S. , Herman W. H. , Zhang P. , Bommer C. , Kuo S. , Boyko E. J. , and Magliano D. J. , IDF Diabetes Atlas: Global, Regional and Country-Level Diabetes Prevalence Estimates for 2021 and Projections for 2045, Diabetes Research and Clinical Practice. (2022) 183, 109119, 10.1016/j.diabres.2021.109119, 34879977. 34879977 PMC11057359

[2] Gupta S. , Dominguez M. , and Golestaneh L. , Diabetic Kidney Disease: An Update, The Medical Clinics of North America. (2023) 107, no. 4, 689–705, 10.1016/j.mcna.2023.03.004. 37258007

[3] Kanwar Y. S. , Sun L. , Xie P. , Liu F. Y. , and Chen S. , A Glimpse of Various Pathogenetic Mechanisms of Diabetic Nephropathy, Annual Review of Pathology. (2011) 6, 395–423, 10.1146/annurev.pathol.4.110807.092150, 2-s2.0-79751477368, 21261520. PMC3700379 21261520

[4] Jin Q. , Liu T. , Qiao Y. , Liu D. , Yang L. , Mao H. , Ma F. , Wang Y. , Peng L. , and Zhan Y. , Oxidative Stress and Inflammation in Diabetic Nephropathy: Role of Polyphenols, Frontiers in Immunology. (2023) 14, 1185317, 10.3389/fimmu.2023.1185317, 37545494. 37545494 PMC10401049

[5] Wada J. and Makino H. , Inflammation and the Pathogenesis of Diabetic Nephropathy, Clinical Science (London, England: 1979). (2013) 124, no. 3, 139–152, 10.1042/CS20120198, 2-s2.0-84872041647. 23075333

[6] Liu H. , Sridhar V. S. , Boulet J. , Dharia A. , Khan A. , Lawler P. R. , and Cherney D. Z. I. , Cardiorenal Protection With SGLT2 Inhibitors in Patients With Diabetes Mellitus: From Biomarkers to Clinical Outcomes in Heart Failure and Diabetic Kidney Disease, Metabolism: Clinical and Experimental. (2022) 126, 154918, 10.1016/j.metabol.2021.154918, 34699838. 34699838

[7] Wang E. , Wang L. , Ding R. , Zhai M. , Ge R. , Zhou P. , Wang T. , Fang H. , Wang J. , and Huang J. , Astragaloside IV Acts Through Multi-Scale Mechanisms to Effectively Reduce Diabetic Nephropathy, Pharmacological Research. (2020) 157, 104831, 10.1016/j.phrs.2020.104831, 32339782. 32339782

[8] Hu Q. , Jiang L. , Yan Q. , Zeng J. , Ma X. , and Zhao Y. , A Natural Products Solution to Diabetic Nephropathy Therapy, Pharmacology & Therapeutics. (2023) 241, 108314, 10.1016/j.pharmthera.2022.108314, 36427568. 36427568

[9] Yuan S. , Zuo B. , Zhou S. C. , Wang M. , Tan K. Y. , Chen Z. W. , and Cao W. F. , Integrating Network Pharmacology and Experimental Validation to Explore the Pharmacological Mechanism of Astragaloside IV in Treating Bleomycin-Induced Pulmonary Fibrosis, Drug Design, Development and Therapy. (2023) 17, 1289–1302, 10.2147/DDDT.S404710, 37138582. 37138582 PMC10150770

[10] Feng H. , Zhu X. , Tang Y. , Fu S. , Kong B. , and Liu X. , Astragaloside IV Ameliorates Diabetic Nephropathy in Db/Db Mice by Inhibiting NLRP3 Inflammasome-Mediated Inflammation, International Journal of Molecular Medicine. (2021) 48, no. 2, 10.3892/ijmm.2021.4996, 34278447. PMC8262660 34278447

[11] Mao Q. , Chen C. , Liang H. , Zhong S. , Cheng X. , and Li L. , Astragaloside IV Inhibits Excessive Mesangial Cell Proliferation and Renal Fibrosis Caused by Diabetic Nephropathy via Modulation of the TGF-*β*1/Smad/miR-192 Signaling Pathway, Experimental and Therapeutic Medicine. (2019) 18, no. 4, 3053–3061, 10.3892/etm.2019.7887, 31572545. 31572545 PMC6755437

[12] Boezio B. , Audouze K. , Ducrot P. , and Taboureau O. , Network-Based Approaches in Pharmacology, Molecular Informatics. (2017) 36, no. 10, 10.1002/minf.201700048, 2-s2.0-85022208333. 28692140

[13] Zhang R. , Zhu X. , Bai H. , and Ning K. , Network Pharmacology Databases for Traditional Chinese Medicine: Review and Assessment, Frontiers in Pharmacology. (2019) 10, 10.3389/fphar.2019.00123, 2-s2.0-85065698881. PMC6393382 30846939

[14] Fotis C. , Antoranz A. , Hatziavramidis D. , Sakellaropoulos T. , and Alexopoulos L. G. , Network-Based Technologies for Early Drug Discovery, Drug Discovery Today. (2018) 23, no. 3, 626–635, 10.1016/j.drudis.2017.12.001, 2-s2.0-85040133166, 29294361. 29294361

[15] Tang G. , Du Y. , Guan H. , Jia J. , Zhu N. , Shi Y. , Rong S. , and Yuan W. , Butyrate Ameliorates Skeletal Muscle Atrophy in Diabetic Nephropathy by Enhancing Gut Barrier Function and FFA2-Mediated PI3K/Akt/mTOR Signals, British Journal of Pharmacology. (2022) 179, no. 1, 159–178, 10.1111/bph.15693, 34638162. 34638162

[16] Hu Y. , Tang W. , Liu W. , Hu Z. , and Pan C. , Astragaloside IV Alleviates Renal Tubular Epithelial-Mesenchymal Transition via CX3CL1-RAF/MEK/ERK Signaling Pathway in Diabetic Kidney Disease, Drug Design, Development and Therapy. (2022) 16, 1605–1620, 10.2147/DDDT.S360346, 35669284. 35669284 PMC9166910

[17] Li X. , Wei S. , Niu S. , Ma X. , Li H. , Jing M. , and Zhao Y. , Network Pharmacology Prediction and Molecular Docking-Based Strategy to Explore the Potential Mechanism of Huanglian Jiedu Decoction Against Sepsis, Computers in Biology and Medicine. (2022) 144, 105389, 10.1016/j.compbiomed.2022.105389, 35303581. 35303581

[18] Li H. , Wang B. , Wu C. , Xie D. , Li J. , Wang N. , Chen H. , and Liu L. , Colquhounia Root Tablet Promotes Autophagy and Inhibits Apoptosis in Diabetic Nephropathy by Suppressing CD36 Expression In Vivo and In Vitro, Journal of Diabetes Research. (2023) 2023, 4617653, 10.1155/2023/4617653, 37622127. 37622127 PMC10447140

[19] Navarro-González J. F. , Jarque A. , Muros M. , Mora C. , and García J. , Tumor Necrosis Factor-Alpha as a Therapeutic Target for Diabetic Nephropathy, Cytokine & Growth Factor Reviews. (2009) 20, no. 2, 165–173, 10.1016/j.cytogfr.2009.02.005, 2-s2.0-63749131183. 19251467

[20] Giunti S. , Barit D. , and Cooper M. E. , Diabetic Nephropathy: From Mechanisms to Rational Therapies, Minerva Medica. (2006) 97, no. 3, 241–262, 16855519. 16855519

[21] Gao W. Y. , Tian M. Y. , Li M. L. , Gao S. R. , Wei X. L. , Gao C. , Zhou Y. Y. , Li T. , Wang H. J. , Bian B. L. , Si N. , Zhao W. , and Zhao H. Y. , Study on the Potential Mechanism of Qingxin Lianzi Yin Decoction on Renoprotection in Db/Db Mice via Network Pharmacology and Metabolomics, Phytomedicine: International Journal of Phytotherapy and Phytopharmacology. (2024) 126, 155222, 10.1016/j.phymed.2023.155222, 38382279. 38382279

[22] Coimbra T. M. , Janssen U. , Gröne H. J. , Ostendorf T. , Kunter U. , Schmidt H. , Brabant G. , and Floege J. , Early Events Leading to Renal Injury in Obese Zucker (Fatty) Rats With Type II Diabetes, Kidney International. (2000) 57, no. 1, 167–182, 10.1046/j.1523-1755.2000.00836.x, 2-s2.0-0033998369, 10620198. 10620198

[23] Zhao Z. H. , Xu M. , Fu C. , Huang Y. , Wang T. H. , Zuo Z. F. , and Liu X. Z. , A Mechanistic Exploratory Study on the Therapeutic Efficacy of Astragaloside IV Against Diabetic Retinopathy Revealed by Network Pharmacology, Frontiers in Pharmacology. (2022) 13, 903485, 10.3389/fphar.2022.903485, 35814228. 35814228 PMC9257082

[24] Shen Q. , Fang J. , Guo H. , Su X. , Zhu B. , Yao X. , Wang Y. , Cao A. , Wang H. , and Wang L. , Astragaloside IV Attenuates Podocyte Apoptosis Through Ameliorating Mitochondrial Dysfunction by Up-Regulated Nrf2-ARE/TFAM Signaling in Diabetic Kidney Disease, Free Radical Biology & Medicine. (2023) 203, 45–57, 10.1016/j.freeradbiomed.2023.03.022, 37030337. 37030337

[25] Fan Y. , Fan H. , Zhu B. , Zhou Y. , Liu Q. , and Li P. , Astragaloside IV Protects Against Diabetic Nephropathy via Activating eNOS in Streptozotocin Diabetes-Induced Rats, BMC complementary and alternative medicine. (2019) 19, no. 1, 10.1186/s12906-019-2728-9, 31805910. PMC6896771 31805910

[26] Catania J. M. , Chen G. , and Parrish A. R. , Role of Matrix Metalloproteinases in Renal Pathophysiologies, American Journal of Physiology. Renal physiology. (2007) 292, no. 3, F905–F911, 10.1152/ajprenal.00421.2006, 2-s2.0-33847788654. 17190907

[27] Khokhar M. , Roy D. , Bajpai N. K. , Bohra G. K. , Yadav D. , Sharma P. , and Purohit P. , Metformin Mediates MicroRNA-21 Regulated Circulating Matrix Metalloproteinase-9 in Diabetic Nephropathy: An In-Silico and Clinical Study, Archives of Physiology and Biochemistry. (2023) 129, no. 6, 1200–1210, 10.1080/13813455.2021.1922457, 34087084. 34087084

[28] Yang X. H. , Feng S. Y. , Yu Y. , and Liang Z. , Study on the Relationship Between the Methylation of the MMP-9 Gene Promoter Region and Diabetic Nephropathy, Endokrynologia Polska. (2018) 69, no. 3, 269–275, 10.5603/EP.a2018.0029, 2-s2.0-85055837477, 29952417. 29952417

[29] Rovira-Llopis S. , Rocha M. , Falcon R. , de Pablo C. , Alvarez A. , Jover A. , Hernandez-Mijares A. , and Victor V. M. , Is Myeloperoxidase a Key Component in the ROS-Induced Vascular Damage Related to Nephropathy in Type 2 Diabetes?, Antioxidants & Redox Signaling. (2013) 19, no. 13, 1452–1458, 10.1089/ars.2013.5307, 2-s2.0-84886289342, 23521574. 23521574 PMC3797450

[30] Zhang C. , Yang J. , and Jennings L. K. , Leukocyte-Derived Myeloperoxidase Amplifies High-Glucose--Induced Endothelial Dysfunction Through Interaction With High-Glucose--Stimulated, Vascular Non--Leukocyte-Derived Reactive Oxygen Species, Diabetes. (2004) 53, no. 11, 2950–2959, 10.2337/diabetes.53.11.2950, 2-s2.0-7044286198. 15504976

[31] Nessler K. , Grzybczak R. , Nessler M. , Zalewski J. , Gajos G. , and Windak A. , Associations Between Myeloperoxidase and Paraoxonase-1 and Type 2 Diabetes in Patients With Ischemic Heart Disease, BMC Cardiovascular Disorders. (2022) 22, no. 1, 10.1186/s12872-022-02928-8, 36463116. PMC9719221 36463116

[32] Mansoor G. , Tahir M. , Maqbool T. , Abbasi S. Q. , Hadi F. , Shakoori T. A. , Akhtar S. , Rafiq M. , Ashraf M. , and Ullah I. , Increased Expression of Circulating Stress Markers, Inflammatory Cytokines and Decreased Antioxidant Level in Diabetic Nephropathy, Medicina (Kaunas, Lithuania). (2022) 58, no. 11, 10.3390/medicina58111604, 36363561. PMC9694611 36363561

[33] Meng X. M. , Nikolic-Paterson D. J. , and Lan H. Y. , Inflammatory Processes in Renal Fibrosis, Nature Reviews. Nephrology. (2014) 10, no. 9, 493–503, 10.1038/nrneph.2014.114, 2-s2.0-84906934479. 24981817

[34] Feigerlová E. and Battaglia-Hsu S. F. , IL-6 Signaling in Diabetic Nephropathy: From Pathophysiology to Therapeutic Perspectives, Cytokine & Growth Factor Reviews. (2017) 37, 57–65, 10.1016/j.cytogfr.2017.03.003, 2-s2.0-85016629235, 28363692. 28363692

[35] Chang W. T. , Huang M. C. , Chung H. F. , Chiu Y. F. , Chen P. S. , Chen F. P. , Lee C. Y. , Shin S. J. , Hwang S. J. , Huang Y. F. , and Hsu C. C. , Interleukin-6 Gene Polymorphisms Correlate With the Progression of Nephropathy in Chinese Patients With Type 2 Diabetes: A Prospective Cohort Study, Diabetes Research and Clinical Practice. (2016) 120, 15–23, 10.1016/j.diabres.2016.07.013, 2-s2.0-84980398239, 27500547. 27500547

[36] Lopez-Castejon G. and Brough D. , Understanding the Mechanism of IL-1*β* Secretion, Cytokine & Growth Factor Reviews. (2011) 22, no. 4, 189–195, 10.1016/j.cytogfr.2011.10.001, 2-s2.0-81155134753, 22019906. 22019906 PMC3714593

[37] Yuan Y. , Li L. , Wang X. , Zhang P. , Wang J. , and Xiao Y. , Correlation Between Plasma NLRP3, IL-1*β*, and IL-18 and Diabetic Nephropathy in Patients With Type 2 Diabetes, Alternative therapies in health and medicine. (2023) 29, no. 4, 52–56, 36947659. 36947659

[38] Lei Y. , Devarapu S. K. , Motrapu M. , Cohen C. D. , Lindenmeyer M. T. , Moll S. , Kumar S. V. , and Anders H. J. , Interleukin-1*β* Inhibition for Chronic Kidney Disease in Obese Mice With Type 2 Diabetes, Frontiers in Immunology. (2019) 10, 10.3389/fimmu.2019.01223, 2-s2.0-85068128221, 31191559. PMC6549251 31191559

