# Supplementary figures and images for: Study on the Potential Mechanism of Astragaloside IV on Renoprotection in Db/Db Mice via Network Pharmacology and Experimental Validation
Source: J Diabetes Res. 2026 Mar 31;2026:5345971. doi: 10.1155/jdr/5345971 (PMC13140807; doi:10.1155/jdr/5345971)

Suppl Fig. 1 WGCNA analysis co-expression modules

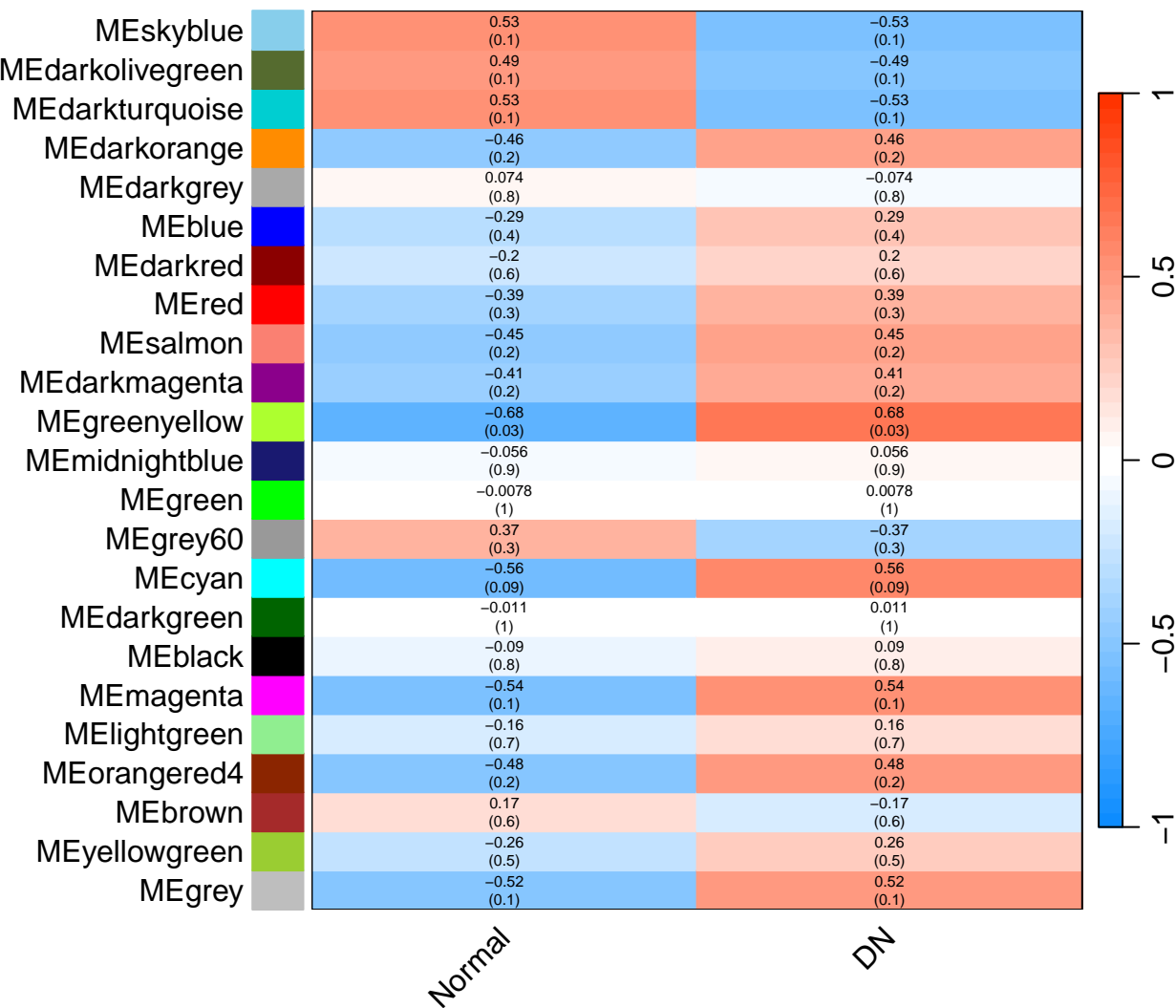

Supplement: Supplementary file 1 — Supporting Information 1 Figure S1: WGCNA coexpression modules. [file JDR-2026-5345971-s001.pdf]
