# Supplementary material for: Study on the Potential Mechanism of Astragaloside IV on Renoprotection in Db/Db Mice via Network Pharmacology and Experimental Validation
Source: J Diabetes Res. 2026 Mar 31;2026:5345971. doi: 10.1155/jdr/5345971 (PMC13140807; doi:10.1155/jdr/5345971)
Supplement: Supplementary file 2 — Supporting Information 2 Table S1: ROC curve quantitative data of core targets. [file JDR-2026-5345971-s002.pdf]

Suppl Tab. 1 ROC curve quantitative data

Target Gene	AUC(95%CI)	Sensitivity(%)	Specificity(%)
MMP-9	1.000(1.000-1.000)	100.00	100.00
MPO	0.920(0.835-1.000)	86.96	90.00
IL6	1.000(1.000-1.000)	100.00	100.00
IL-1β	1.000(1.000-1.000)	100.00	100.00
